# Complete chloroplast genome of *Disporum sessile*

**DOI:** 10.1080/23802359.2019.1698993

**Published:** 2019-12-13

**Authors:** Inkyu Park, Goya Choi, Sungyu Yang

**Affiliations:** Herbal Medicine Research Division, Korea Institute of Oriental Medicine, Naju, Republic of Korea

**Keywords:** *Disporum sessile*, medicinal plant, chloroplast genome, Colchicaceae

## Abstract

*Disporum sessile* roots are used as a medicinal herb. To ensure the accurate identification of *D. sessile*, we sequenced its chloroplast genome using the Illumina MiSeq platform. Results showed that the chloroplast genome of *D. sessile* is 159,102 bp in size, with a large single-copy (LSC) region (85,456 bp), a small single-copy (SSC) region (17,108 bp), and two inverted repeat (IR) regions (IRa and IRb; 28,269 bp each). Additionally, the chloroplast genome contains 112 unique genes, including 78 protein-coding, four ribosomal RNA, and 30 transfer RNA genes. Phylogenetic analysis revealed that *D. sessile* is most closely related to *Colchicum autumnale*.

*Disporum sessile* (Thunb.) D.Don ex Schult. & Schult.f. (Colchicaceae) is a subendemic species that is restrictively distributed in South Korea, Japan, and the Sakhalin Island of Far East Russia (Lee 2007; Tamura 2016). *Disporum sessile* is morphologically similar to *D. uniflorum*, and both species are occasionally considered as conspecies (Lee 1979, 1996). Additionally, many infraspecies are taxonomically categorized under these two species (The Plant List, 2013). Roots of *D. sessile* have been traditionally used as a medicinal herb (Korea Institute of Oriental Medicine (KIOM) 2019), and their antioxidant and estrogenic activities have been reported previously (Williams et al. 1993; Jeon et al. 2010). However, morphological similarities between *D. sessile* and *D. uniflorum* and problems with taxonomical classification negatively affect the use of *D. sessile*. In this study, we aimed to resolve these taxonomical issues and ensure the accurate identification of *D. sessile*. Therefore, we sequenced and analyzed the complete chloroplast genome of *D. sessile*. Our data provide fundamental information that will assist in the continued use of *D. sessile* as a herbal medicine.

Fresh leaves of *D. sessile* were collected from its native habitat in Korea (33°23′55.7″N and 126°25′26.8″E). Specimens were labeled with unique identification numbers and registered in the Korean Herbarium of Standard Herbal Resources (Index herbariorum code: KIOM) at the Korea Institute of Oriental Medicine (KIOM), with the voucher number KIOM201901022378. Genomic DNA was extracted from leaf samples using the DNeasy Plant Maxi Kit (QIAGEN, Valencia, CA). An Illumina paired-end library was constructed and sequenced using the MiSeq platform (Illumina Inc., San Diego, CA). The complete chloroplast genome of *D. sessile* was deposited in the GenBank database of the National Center for Biotechnology Information (NCBI) under the accession number MN332241.

Illumina sequencing of *D. sessile* chloroplast genome yielded 2.4 Gb of high-quality paired-end reads. The chloroplast genome sequence contigs of *D. sessile* were *de novo* assembled, based on comparison with low-coverage whole-genome sequences (Kim et al. 2015). Results showed that the complete chloroplast genome of *D. sessile* is 159,102 bp in length, with a typical quadripartite structure comprising a large single-copy (LSC) region of 85,456 bp, a small single-copy (SSC) region of 17,107 bp, and two inverted repeat (IR) regions, IRa and IRb, of 28,269 bp each. The GC content of the chloroplast genome was 37.3%, with the IR regions showing a higher GC content (42%) than the LSC (35.5%) and SSC (31%) regions. These data indicate that the chloroplast genome of *D. sessile* is AT-rich, which is consistent with the chloroplast genomes of other plant species (Park et al. 2017). The chloroplast genome of *D. sessile* harbored 112 unique genes, including 78 protein-coding genes, 30 transfer RNA (tRNA) genes, and four ribosomal RNA (rRNA) genes. Of the 112 genes, 17 were duplicated in the IR regions, and 18 contained introns. Among the 18 intron-containing genes, 16 contained a single intron, and two (*ycf3* and *clpP*) harbored two introns. We also analyzed simple sequence repeats (SSRs) and tandem repeats in the chloroplast genome sequence of *D. sessile*. A total of 60 SSRs were detected in intergenic regions, and most of these contained mono- and dinucleotide repeats. Additionally, 22 tandem repeats (>20 bp) were detected in intergenic and exonic regions; the *ycf1* gene contained the highest number of tandem repeats

To investigate the phylogenetic relationship of *D. sessile* with other plant species, we aligned the nucleotide sequences of 58 protein-coding genes of *D. sessile* and their homologs from 10 other taxa, spanning a total length of 56,272 bp. The phylogenetic tree, constructed using the maximum-likelihood (ML) method, contained 12 nodes, each with a bootstrap value of 100% (Figure 1). *Disporum sessile* showed a strong phylogenetic relationship with the Liliales order. Additionally, *D. sessile* formed a monophyletic group with *Colchicum autumnale* within Colchicaceae, with 100% bootstrap values (Figure 1).

**Figure 1.**
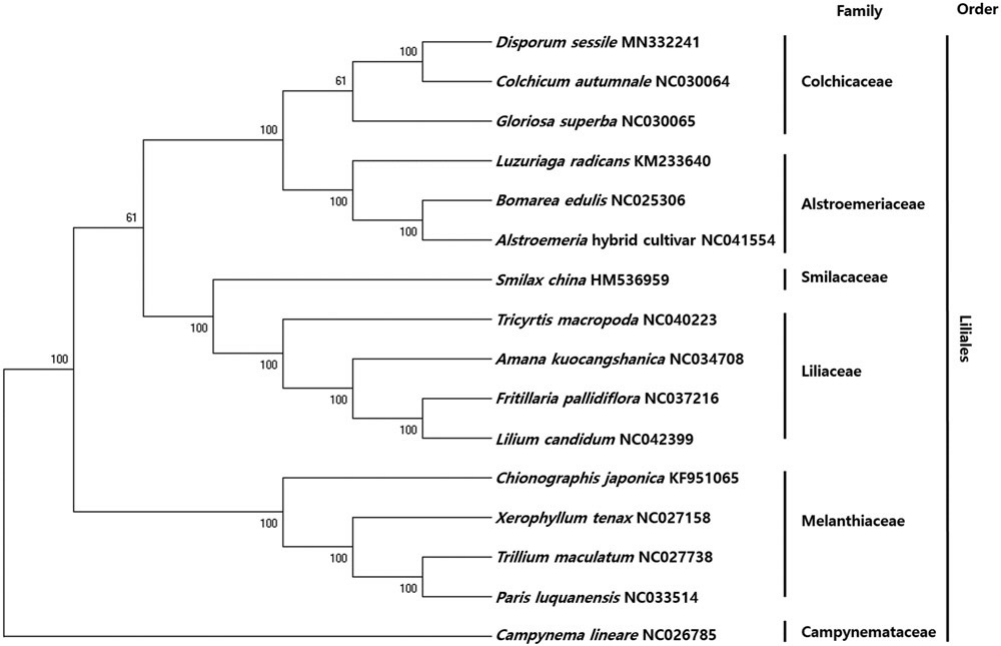
Phylogenetic analysis of 16 taxa, including *Disporum sessile* and one outgroup taxon, based on chloroplast protein-coding genes. Nucleotide sequences of 58 protein-coding genes were aligned using MAFFT (Katoh et al. 2002). The phylogenetic tree was constructed using the maximum likelihood (ML) method in MEGA6, with 1,000 bootstrap replicates (Tamura et al. 2013). Bootstrap values are indicated at the nodes.
